# Barriers to Utilizing Medicaid Smoking Cessation Benefits

**Published:** 2017-11-30

**Authors:** Blaine Knox, Scott Mitchell, Ellen Hernly, Alicia Rose, Hilary Sheridan, Edward F. Ellerbeck

**Affiliations:** University of Kansas School of Medicine, Department of Preventive Medicine and Public Health, Kansas City, KS

**Keywords:** smoking cessation, Medicaid, tobacco use cessation products

## Abstract

**Introduction:**

Smoking is the number one preventable cause of death in the United States. Under the Affordable Care Act, Kansas Medicaid covers all seven FDA-approved smoking cessation therapies. However, it is estimated only 3% of Kansas Medicaid smokers use treatment compared to the national estimate of 10%. The objective is to determine systemic barriers in place that prevent optimal utilization of Medicaid smoking cessation benefits among KU Medical Center Internal Medicine patients

**Methods:**

For this quality improvement project, a population of 169 Kansas Medicaid smokers was identified who had been seen at the KU Internal Medicine Clinic from January 1, 2015 – February 16, 2016. Phone surveys were completed with 62 individuals about smoking status, interest in using smoking cessation treatment options, and awareness of Medicaid coverage of treatment.

**Results:**

Of the 62 respondents, 24 (39%) were prescribed pharmacotherapy and 41 (66%) were interested in using smoking cessation treatment. There were eight who had quit smoking. Of the remaining 54 smokers, 31 (57%) were unaware that Medicaid would cover pharmacotherapy. Of 24 participants who received a prescription for pharmacotherapy, 13 (54%) were able to fill the prescription at no cost using the Medicaid benefit.

**Conclusions:**

The majority of respondents were interested in using smoking cessation treatment yet three main barriers existed to using Medicaid smoking cessation benefits: physicians not prescribing treatment to patients, patients not aware of Medicaid coverage, and inadequate pharmacy filling. Improved physician and patient awareness of Medicaid coverage will facilitate more patients receiving smoking cessation therapy and ultimately quitting smoking.

## Introduction

Smoking is the leading preventable cause of death in the United States.^1^ Medicaid enrollees are twice as likely to be smokers as the general population, 32% vs 17%,^2^ which places a large financial burden on the Medicaid program. The cost of smoking-related disease in Medicaid patients is estimated to be more than $75 billion which is about 15% of all Medicaid expenditures. Many smokers want to quit and there are a variety of options available to them. Evidence-based tobacco dependence treatments (TDT) include individual, group, and telephone counseling, along with seven FDA-approved nicotine replacement therapies (NRT; nicotine patch, gum, lozenge, nasal spray and inhaler, bupropion (Zyban), and varenicline (Chantix)).^2^–^4^ Despite these options for treatment, Kansas has performed poorly compared to national NRT usage since passage of the ACA. In 2013, 49,000 (35%) of Kansas Medicaid enrollees were smokers with only 3% of those using medications.^2^ Additionally, the rate of NRT utilization in Kansas from 2011–2013 was 0.05 prescriptions per smoker, compared with 0.20 prescriptions per smoker nationally.^2^ These data placed Kansas 48th out of 50 states in terms of the frequency with which Medicaid smokers receive NRT.

Medicaid smokers often do not get help quitting due to multiple barriers including cost of treatments, prior authorization requirements, lack of awareness of options amongst Medicaid enrollees and physicians, as well as physician time constraints and perceived patients’ willingness to quit.^5^ As of January 2014, the Affordable Care Act (ACA) required Medicaid programs to cover smoking cessation treatment, including over-the-counter medications.^6^ Previously identified barriers, such as cost to the patient of pharmacotherapy and insurance company resistance to coverage, are negated partially by the Affordable Care Act mandate for Medicaid to cover NRT at no cost to the patient. A systematic review of smoking cessation guidelines recommended that clinicians should encourage all patients interested in quitting to utilize tobacco dependence therapy to aid in cessation unless they are light smokers, adolescents, pregnant women, or smokeless tobacco users.^7^

To bridge the gap between expanded Medicaid coverage and utilization of coverage in a practical sense, the process a patient undergoes to procure and use cessation treatment must be understood. There are many potential pitfalls in the process, including lack of physician and patient knowledge of Medicaid coverage, treatment not being prescribed, lack of pharmacist knowledge of which National Drug Codes cover which specific NRT, confusion at the pharmacy regarding specific product coverage, and patients’ perception of NRT effectiveness and willingness to use.^5^ Indeed, counselors in the tobacco treatment service at the University of Kansas Medical Center reported that some patients were not getting prescriptions for smoking cessation medications and others who had received prescriptions for NRT were not able to get these prescriptions filled at the pharmacy. To better understand this potential problem in the quality of smoking cessation services, we assessed current barriers to treatment from a patient perspective in order to identify which of these represent the principal barrier or barriers to patients obtaining and utilizing the Medicaid smoking cessation benefit and ultimately in quitting smoking.

## Methods

### Participants and setting

**Figure 1:**
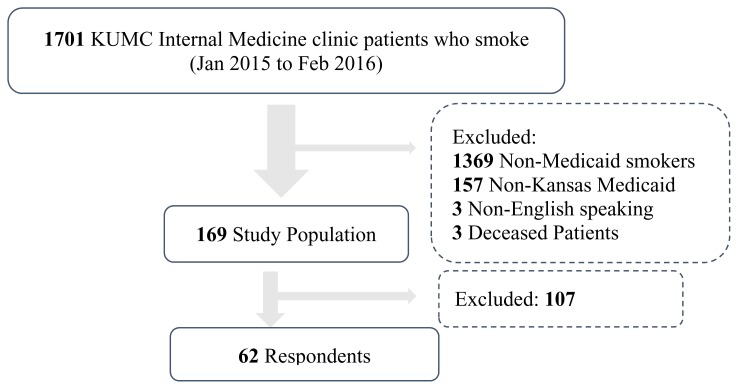
The flowchart depicts how patients were identified and included or excluded.

Using the Heron^9^ system interface to the electronic health record, we identified patients 18 years or older seen in the University of Kansas Medical Center (KUMC) Internal Medicine Clinic between January 1, 2015 and January 1, 2016 who were identified as smokers. From this group, we selected patients who were enrolled in Kansas Medicaid and excluded patients who were deceased or for whom English was not their primary language. One or more attempts were made to contact each of these patients by telephone. Upon reaching a patient by telephone, the patient was provided with a brief verbal description of the project and provided their assent for participation in a brief telephone survey (Figure 1).

### Data Collection

Demographic data were captured from the electronic health record through the HERON interface, including gender, race, and age. People who responded to the phone survey were asked whether they were interested in quitting smoking, counseled by their doctor on the benefit of quitting, interested in using treatment to help them quit, prescribed pharmacotherapy, what type of pharmacotherapy they received, if they filled their prescription, if their prescription was filled at no cost, if they used the prescription, and if the prescription helped them quit. Data collected via interviews were entered into and stored securely using REDCap electronic data capture tools hosted at the University of Kansas Medical Center.^10^

### Data Analysis

The primary outcome was the proportion of KUMC Internal Medicine Medicaid enrollees who received pharmacotherapy for tobacco cessation and were able to utilize the Medicaid benefit. Secondary outcomes included the proportion of Internal Medicine Medicaid enrollees who were counseled about quitting smoking in the last year, the proportion that were prescribed pharmacotherapy, the proportion aware of Medicaid coverage of pharmacotherapy, and the proportion interested in receiving pharmacotherapy in the future. All outcomes were calculated as simple frequencies. Data analysis was conducted using Microsoft Excel after removal of all the protected health information.

This project was reviewed by the KUMC Institutional Review Board and deemed as a quality improvement project designed to improve uptake and utilization of smoking cessation pharmacotherapy.

## Results

### Prescription and Utilization

Of the 169 smokers that met the inclusion criteria, 62 (37%) responded to the survey. The mean age of the survey respondents was 53 years. Approximately half were female (52%) and Caucasian (52%), while 45% were African American (Table 1).

Of the 62 respondents, 41 (66%) were interested in receiving cessation therapy and 24 (39%) of patients had been prescribed NRT. Of the 24 patients prescribed therapy, 20 (83%) filled their prescription at the pharmacy. Of the 20 that filled their prescription, 13 (65%) took advantage of the Medicaid benefit and filled it at no cost. Also, 80% of patients prescribed therapy reported using the therapy and 38% of patients reported that the NRT prescribed aided them in quitting smoking (Table 2). When patients were asked whether they were aware that Kansas Medicaid should cover their prescription therapy only 23 (43%) of patients were aware this option existed.

### Nicotine Replacement Therapy

**Figure 2:**
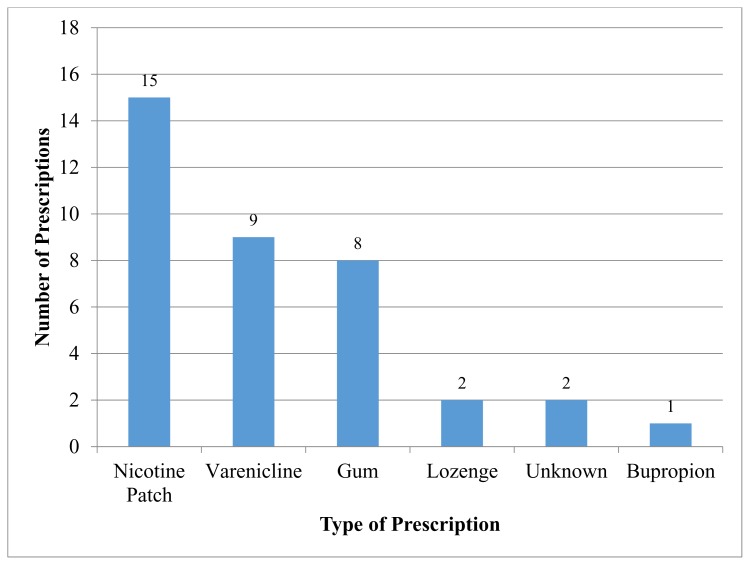
Types of pharmacotherapy prescribed.

The nicotine patch was the number one prescribed tobacco cessation therapy representing 15 out of the 37 (41%) prescribed therapies. Varenicline was prescribed to 24% of patients and the nicotine gum was prescribed to 22% of patients. The nicotine lozenge was prescribed to only 5% of patients and bupropion was prescribed to only 2.7% of patients. Another 2.7% of patients were prescribed therapy but were unsure of the specific therapy their doctor recommended (Figure 2). The majority of prescriptions for tobacco cessation therapy were for the nicotine patch followed by varenicline and the nicotine gum; together these three represent 90% of total prescribed therapies. There were a total of 37 prescriptions for tobacco therapy given to a total of 24 patients as some patients were prescribed multiple therapies.

### Pharmacy Coverage

There was variation between pharmacies on whether prescriptions were filled at no out of pocket cost to patients. Of the 37 prescriptions that were attempted to be filled, 20 (54%) were filled at no cost to the patient thus honoring the Medicaid benefit. Out of the 5 patients who filled their prescription at Walgreen’s, four (80%) took advantage of the Medicaid benefit and paid no out of pocket cost. This is compared to one of the five patients (20%) who went to Walmart who took advantage of the Medicaid benefit. All three patients who filled their prescription at CVS took advantage of the benefit.

## Discussion

Three main barriers existed for Medicaid patients interested in smoking cessation from receiving treatment. First, physicians did not prescribe therapy to all of their patients who expressed interest in quitting smoking. Of the 41 patients interested in receiving therapy, only 24 (58%) were prescribed NRT. This is consistent with previous statistics on low prescribing practices amongst Kansas physicians to Medicaid patients.^2^ Physician lack of prescribing represents the main barrier to patients interested in quitting, however, when physicians prescribe therapy, the nicotine patch and gum along with varenicline are the most frequent choices for therapy. Further studies will be needed to elucidate whether the low prevalence of prescribing practices is due to physician lack of knowledge of Medicaid coverage of NRT or if physician time constraint due to the many underserved areas in Kansas accounts for this statistic.

Another barrier to patients receiving therapy is the fact that only 43% of patients who identify as smokers were aware that Medicaid should cover their NRT. The majority of Medicaid patients were disadvantaged socioeconomically and already struggling with the burden of high medical costs. This lack of awareness that therapy should be covered presents another deterrent to those interested in quitting. Increased physician awareness that their patients are interested in quitting and that Medicaid should cover NRT will foster more conversations with patients about smoking cessation leading to more people quitting.

The third main barrier that was identified was NRT filling by pharmacies and whether patients were able to take advantage of the Medicaid benefit. According to our survey, the rate of prescriptions that were filled at no cost to the patient was 54% with evidence that different pharmacies varied on whether they required patients to pay. A comprehensive assessment of pharmacy filling practices to identify to patients where they should have NRT filled would reduce patient costs and enable them to use the full benefit of Medicaid available to them.

Utilizing Medicaid’s policy on tobacco cessation would cut costs to the program as well as decrease morbidity and mortality to its enrollees. A cost-benefit study in Massachusetts analyzed the financial cost of smoking cessation per patient compared to the reduced financial cost of hospital admissions that smoking cessation provides to the state Medicaid program.^8^ Every $183 spent per patient on tobacco cessation averted an average of $571 per patient on hospital admissions, equivalent to $2.21 saved for each $1 spent. This is important as it shows that states that invest in cessation therapy avoid long term costs from increased patient morbidity and hospitalizations incurred from smoking. While the reduced cost to the system is one benefit, more importantly, patients who quit live healthier lives with less disease burden.^1^

There are several limitations to this study. First, surveys were self-reported by patients which required them to remember a conversation with their provider that could have been a year earlier. Also, patients were sampled from one tertiary care facility in Kansas which may not be representative of all Medicaid patients across the state. The response rate for this study was 37% which may not reflect the entire population; however, the demographics were similar between those who responded to the phone interview and the study population as a whole.

The results of the project have strong potential to direct future care of Medicaid enrollees who smoke. This information helps providers understand the state of cessation therapy being prescribed to this patient population in Kansas and inform future improvements in prescribing practices. It is also important for Medicaid patients to be aware that therapy should be covered and they should try to fill their prescription at certain pharmacies and try multiple pharmacies before paying out of pocket. Some of the specific future interventions could include patient education through pamphlets at clinics, increased physician awareness of coverage and patient interest, and modifications of the electronic medical record to facilitate conversations in the clinic about smoking cessation therapy.

## Figures and Tables

**Table 1:** Demographics of population studied.

Male	30 (48%)
Female	32 (52%)
Caucasian	32 (52%)
African American	20 (45%)

**Table 2:** Results of phone survey (n; %).

Counseled by Doctor on Quitting	57 (92%)
Interested in Quitting	47 (76%)
Interested in Therapy	41 (65%)
Prescribed Therapy	24 (39%)
Filled Prescription	20 (32%)
Filled Prescription at no Cost	13 (65%)
Used Prescription	15 (24%)
Prescription Aided in Quitting	9 (15%)

## References

[1] US Department of Health and Human Services (2014). The Health Consequences of Smoking – 50 Years of Progress: A Report of the Surgeon General.

[2] Ku L, Bruen BK, Steinmetz E, Bysshe T (2016). Medicaid tobacco cessation: Big gaps remain in efforts to get smokers to quit. Health Affairs.

[3] Singleterry J, Jump Z, DiGiulio A (2015). State Medicaid coverage for tobacco cessation treatments and barriers to coverage - United States, 2014–2015. MMWR Morb Mortal Wkly Rep.

[4] Cahill K, Stevens S, Perera R, Lancaster T (2013). Pharmacological interventions for smoking cessation: An overview and network meta-analysis. Cochrane Database Syst Rev.

[5] Sheffer C, Anders M, Brackman SL, Steinberg MB, Barone C (2012). Tobacco intervention practices of primary care physicians treating lower socioeconomic status patients. Am J Med Sci.

[6] US Centers for Disease Control and Prevention (2014). Smoking and Tobacco Use: State Highlights: Kansas.

[7] Fiore MC, Jaén CR, Baker TB (2008). Treating Tobacco Use and Dependence: 2008 Update. Clinical Practice Guideline.

[8] Richard P, West K, Ku L (2012). The return on investment of a Medicaid tobacco cessation program in Massachusetts. PLoS One.

[9] Waitman LR, Warren JJ, Manos EL, Connolly DW (2011). Expressing Observations from Electronic Medical Record Flowsheets in an i2b2 based Clinical Data Repository to Support Research and Quality Improvement. AMIA Annu Symp Proc.

[10] Harris Paul A, Taylor Robert, Thielke Robert, Payne Jonathon, Gonzalez Nathaniel, Conde Jose G (2009). Research electronic data capture (REDCap) – A metadata-driven methodology and workflow process for providing translational research informatics support. J Biomed Inform.

